# An anthocyanin rich strawberry extract induces apoptosis and ROS while decreases glycolysis and fibrosis in human uterine leiomyoma cells

**DOI:** 10.18632/oncotarget.15333

**Published:** 2017-02-15

**Authors:** Md Soriful Islam, Francesca Giampieri, Milijana Janjusevic, Massimiliano Gasparrini, Tamara Y. Forbes-Hernandez, Luca Mazzoni, Stefania Greco, Stefano Raffaele Giannubilo, Andrea Ciavattini, Bruno Mezzetti, Franco Capocasa, Mario Castellucci, Maurizio Battino, Pasquapina Ciarmela

**Affiliations:** ^1^ Department of Experimental and Clinical Medicine, Università Politecnica delle Marche, Ancona, Italy; ^2^ Biotechnology and Microbiology Laboratory, Department of Botany, University of Rajshahi, Rajshahi, Bangladesh; ^3^ Department of Clinical Science, Università Politecnica delle Marche, Ancona, Italy; ^4^ Department of Agricultural, Food and Environmental Sciences, Università Politecnica delle Marche, Ancona, Italy; ^5^ Department of Information Engineering, Università Politecnica delle Marche, Ancona, Italy

**Keywords:** strawberry, uterine leiomyoma, fibrosis, apoptosis, ROS

## Abstract

Uterine leiomyomas are highly prevalent benign tumors in reproductive aged women. Unfortunately, medical treatments are still limited and no preventive therapies have been developed. In the present study, we investigated the therapeutic effects of strawberry extract on uterine leiomyoma cells. Leiomyoma and myometrial cells were treated with strawberry (cultivar *Alba*) extract (250 μg/ml) for 48 h to measure apoptosis, reactive oxygen species (ROS), oxidative phosphorylation (OCR, oxygen consumption rate) and glycolysis (ECAR, extracellular acidification rate) as well as fibrosis associated gene and/or protein expression. In leiomyoma cells, strawberry increased the percentage of apoptotic and dead cells. Strawberry significantly increased ROS concentration in leiomyoma cells, while decreased it in myometrial cells. After strawberry treatment, leiomyoma cells showed a significant decreased rate of ECAR, while OCR was unchanged in both myometrial and leiomyoma cells. Strawberry significantly decreased collagen1A1, fibronectin and versican mRNA expression in leiomyoma cells. The reduced protein expression of fibronectin was observed by strawberry extract in leiomyoma cells as well. Furthermore, strawberry was able to reduce activin A induced fibronectin, collagen1A1, and versican as well as activin A and PAI-1 mRNA expression in leiomyoma cells. This study suggests that strawberry can be developed as therapeutic and/or preventive agent for uterine leiomyomas.

## INTRODUCTION

Uterine leiomyomas (fibroids or myomas) are benign smooth muscle tumors originating from the myometrium [1]. They are highly prevalent, with ~70% of white women and more than 80% of African-American women by the age of 50 years [2]. Approximately 25% of women shows significant clinical symptoms, such as abnormal uterine bleeding, abdominal pain and discomfort, pregnancy complications and even infertility [1, 3]. This complicated disease exerts an enormous burden on health care system worldwide. Fibroids are the leading indication for hysterectomy, responsible for total direct and indirect annual costs of $5.89-$34.37 billion in United States alone [4].

Although leiomyoma treatment has mainly been surgical some medical therapies are available for women who wish to preserve their uterus. These include gonadotropin-releasing hormone (GnRH) agonists (leuprolide acetate), GnRH antagonists (cetrorelix acetate), selective progesterone receptor modulators (ulipristate acetate and asoprisnil), antiprogestin (mifepristone), etc. [1]. However, they are only partially useful. For example, leuprolide acetate is approved as preoperative short-term therapy for uterine fibroids. It is able to reduce uterine and leiomyoma volume [5] as well as alleviate bleeding and increase hemoglobin levels [6]. However, this treatment frequently causes side effects such as hot flushes, vaginitis, and bone loss, etc. [5]. It is noted that in 2012 ulipristate acetate (5mg) was approved by European Medicines Agency (EMA) for the leiomyoma treatment of moderate to severe symptoms-limited to 3 months and pre-surgery. Therefore, there is an urgent need to develop safe and effective nonsurgical treatments for women with uterine fibroids.

Dietary phytochemicals are non-nutritive compounds with disease-preventive properties, mainly found in fruits, vegetables, cereals, legumes, herbs, spices, nuts and seeds. The ability of dietary phytochemicals to regulate tumorigenic processes (such as, inflammation, fibrosis, proliferation and angiogenesis) has been proven to be key source in modern drug discovery programs [7]. In this context, several dietary phytochemicals have studied in uterine leiomyoma. Among these, epigallocatechin gallate (EGCG), a strong polyphenol catechin found in green tea [*Camellia sinensis* (L.) Kuntze], has shown interesting results during *in vivo* and course of clinical trial. EGCG was reported to dramatically reduce the volume and weight of tumors of female mice (implanted with fibroid tumor cells) [8], and incidence and size of spontaneously occurring leiomyoma of the oviduct in Japanese quail [9]. In addition, green tea extract significantly reduced uterine fibroid volume, fibroid-specific symptom severity, and induced significant improvement in health-related quality-of-life in premenopausal women compared to the placebo group [10]. Furthermore, in a prospective cohort study, it was found that a high intake of fruit, particularly citrus fruit, was inversely associated with uterine fibroids risk among black women [11].

Strawberries are rich of bioactive compounds such as, anthocyanins, flavonols, flavanols, condensed tannins (proanthocyanidins, ellagitannins, and gallotannins), hydroxybenzoic and hydroxycinnamic acid derivatives, and hydrolyzable tannins [12]. Evidence from *in vitro* studies show that strawberries may have antiinflammatory effects [13], and suppress mutagenesis through antioxidative and genoprotective properties [14]. Strawberry was also reported to induce apoptosis [15], and inhibit proliferation of several type of cancer cells [16]. No studies have been performed on the effect of strawberries in uterine cells. In order to investigate if the use of strawberry extracts could be useful for the treatment and/or the prevention of uterine leiomyoma, we tested their efficacy on healthy uterine (myometrial) and on leiomyoma cells.

Therefore, in the present study, we investigated the *in vitro* effect of a variety of strawberry selected for higher sensorial and nutritional quality [17]. Strawberry of *Alba* cultivar [17] extract was used to evaluate the effect on apoptosis, reactive oxygen species (ROS) as well as oxidative phosphorylation and glycolysis in myometrial and leiomyoma cells. Considering the role of fibrosis in leiomyoma growth, we also investigated the effect of strawberry extract on fibrosis associated factors in this cell types.

## RESULTS

### Cytotoxic effects of strawberry extract on myometrial and leiomyoma cells

**Figure 1:**
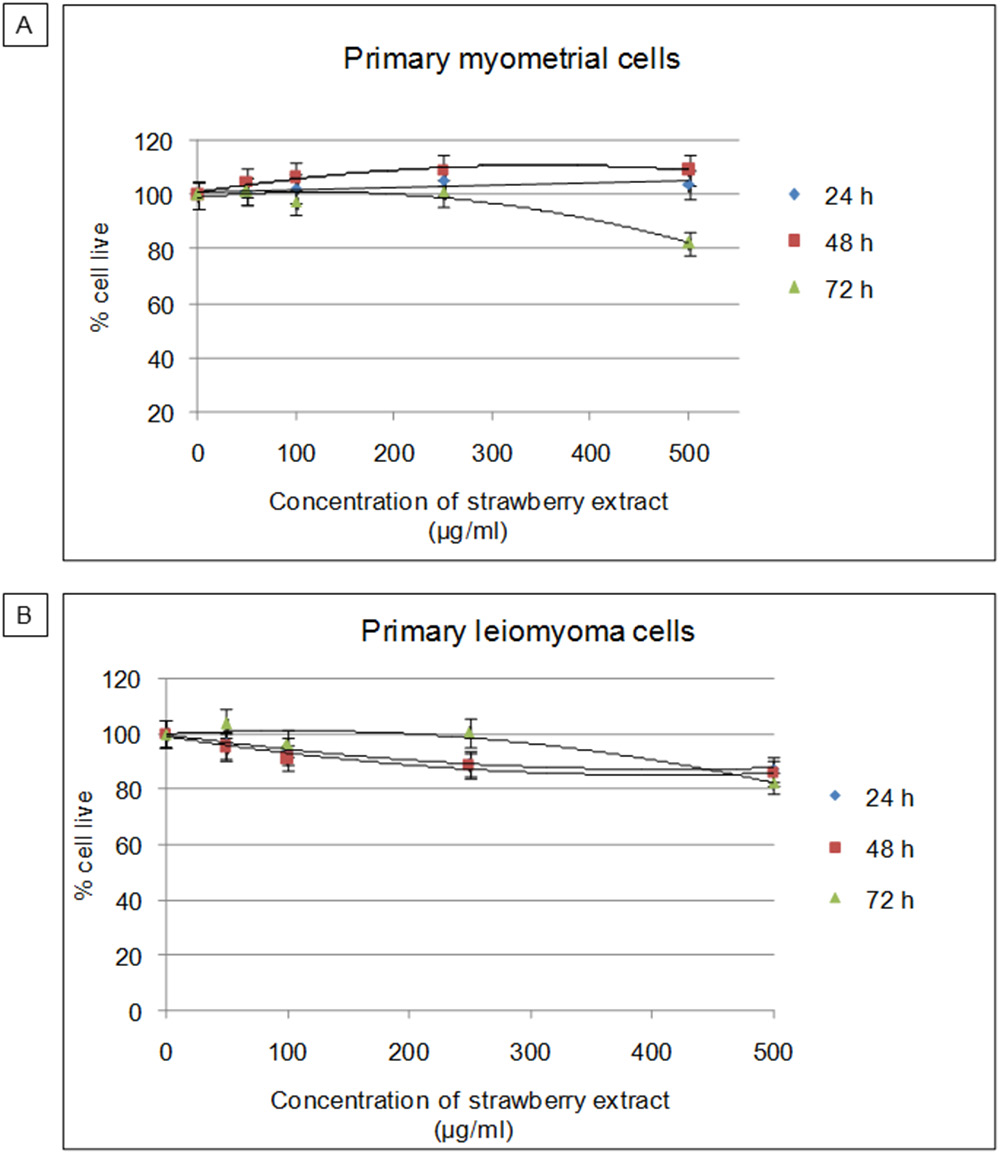
Cytotoxic effects of strawberry extract on myometrial and leiomyoma cells **A**. Viability of myometrial cells. **B**. Viability of leiomyoma cells. MTT assay performed after exposure to increasing concentration of strawberry extract (50, 100, 250, 500 μg/ml) for different incubation times (24 h, 48 h, 72 h). Data are expressed as percentage of live cells. Mean ± SD of three replications.

First, the possible cytotoxic effect of the strawberry extract in relation to increasing concentration and exposure time was studied. Primary myometrial and leiomyoma cells were treated with strawberry extract at different concentrations (50, 100, 250, 500 μg/ml) for 24 h, 48 h and 72 h, and cell viability was determined by MTT assay. As shown in Figure 1, vitality did not vary with increases in strawberry extract concentration or with the exposure time both for myometrial and leiomyoma cells, thereby demonstrating that the extracts were not cytotoxic under the experimental conditions. According to these results, the combination of extract concentration and time of exposure that gave the best results in terms of cell viability and reproducibility (250μg/ml for 48h) was selected for further experiments.

### Effect of strawberry extract on induction of apoptosis in myometrial and leiomyoma cells

**Figure 2:**
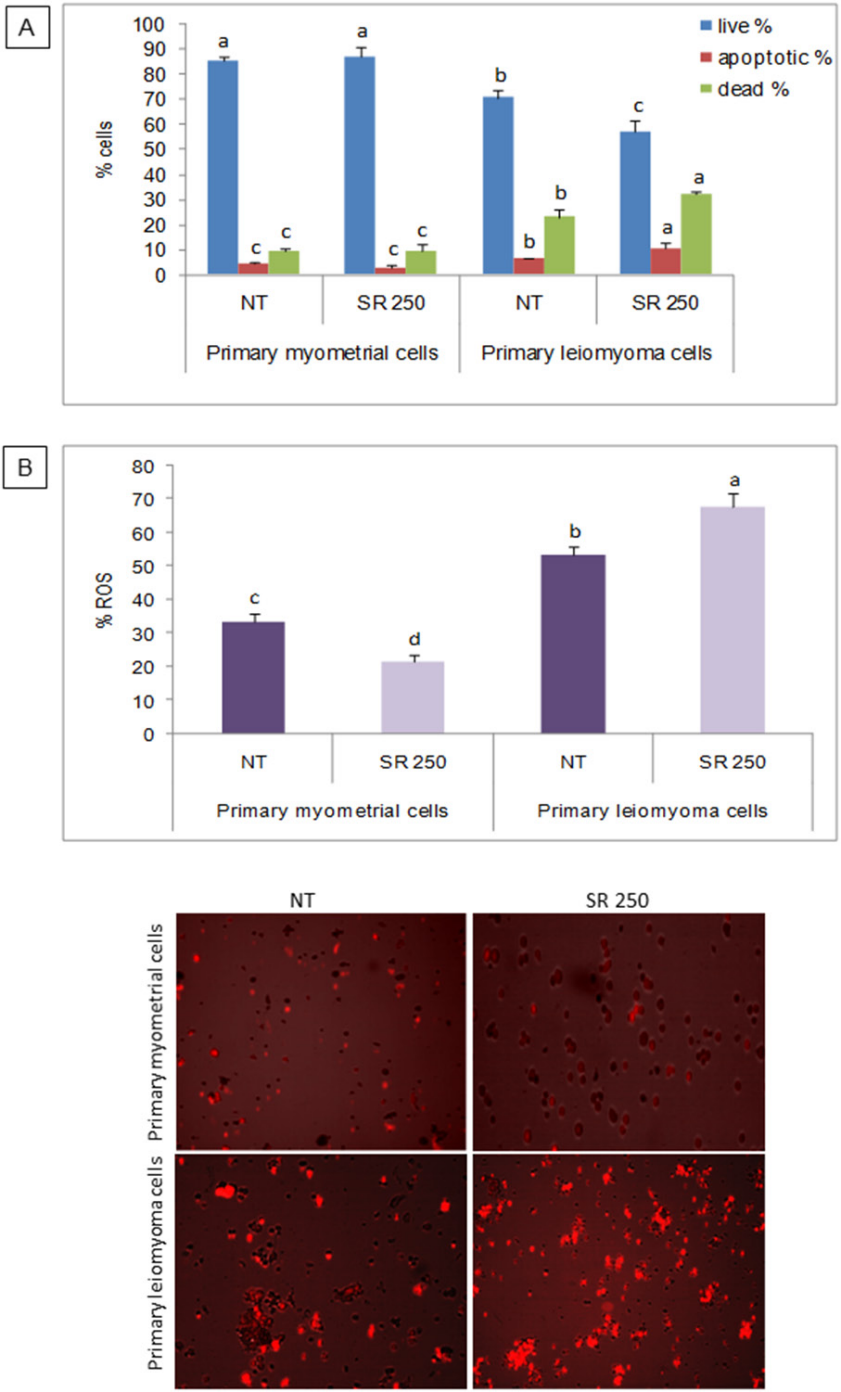
Effect of strawberry extract on induction of apoptosis and ROS in myometrial and leiomyoma cells **A**. Percentage of live, dead and apoptotic cells. **B**. Intracellular ROS concentration. Representative images are shown below the histograms. Columns labeled with different letters are significantly different from the control (P<0.05). Mean ± SD of three replications. NT, no treatment; SR 250, strawberry extract at 250 μg/ml.

In order to evaluate the effect of strawberries in cell viability, myometrial and leiomyoma cells were treated with strawberry extract at 250 μg/ml for 48 h, and the apoptotic/dead/live cell numbers were analysed using the Tali™ apoptosis assay kit. In strawberry treated leiomyoma cells, the percentage of apoptotic and dead cells was significantly higher and live cells were significantly lower in strawberry treated leiomyoma cells compared to untreated control (Figure 2A). Interestingly, the percentage of apoptotic, dead and live cells were not different in strawberry treated myometrial cells compared to untreated cells (Figure 2A).

### Effect of strawberry extract on intracellular ROS concentration in myometrial and leiomyoma cells

To determine the intracellular ROS levels, myometrial and leiomyoma cells were treated with strawberry extract (250 μg/ml) for 48 h, and analyzed using the probe CellROX® Orange reagent. In agreement with apoptosis results, a significant increase (P< 0.05) in ROS levels was found after strawberry treatment in leiomyoma cells (P< 0.05) while in myometrial cells the intracellular ROS concentration was significantly lower after the pre-incubation with strawberry extract (P< 0.05) (Figure 2B).

### Effect of strawberry extract on oxidative phosphorylation and glycolysis in myometrial and leiomyoma cells

**Figure 3:**
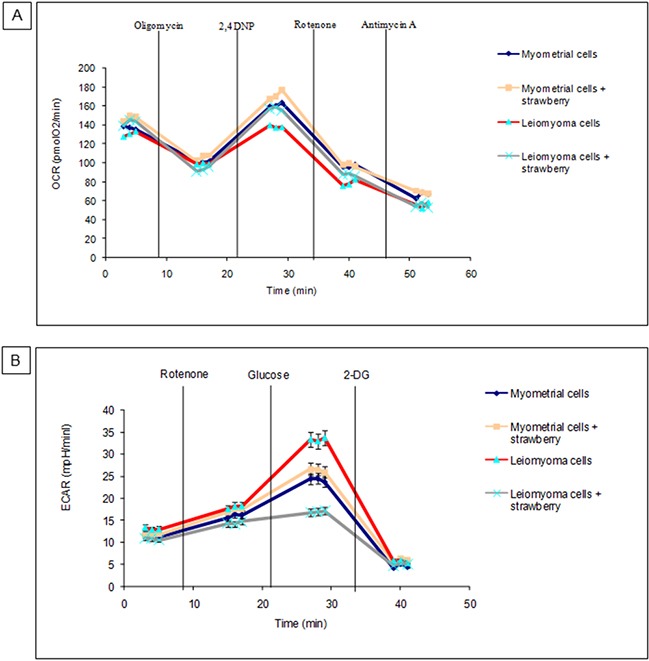
Metabolic characterization of myometrial and leiomyoma cells **A**. OCR or oxidative phosphorylation. **B**. ECAR or glycolysis. Data are presented as mean ± SD of three replications.

To measure oxidative phosphorylation and glycolysis in myometrial and leiomyoma cells, Seahorse XF24 Extracellular Flux Analyzer was employed. We treated myometrial and leiomyoma cells strawberry extract (250 μg/ml) for 48 h, and measured OCR and ECAR. As shown in Figure 3A-3B, both myometrial and leiomyoma cells present an increase in OCR after strawberry treatment, though statistically not significant, while regarding the ECAR leiomyoma cells presented a significant decrease (P< 0.05) respect to the controls.

### Effect of strawberry extract on ECM components expression in myometrial and leiomyoma cells

**Figure 4:**
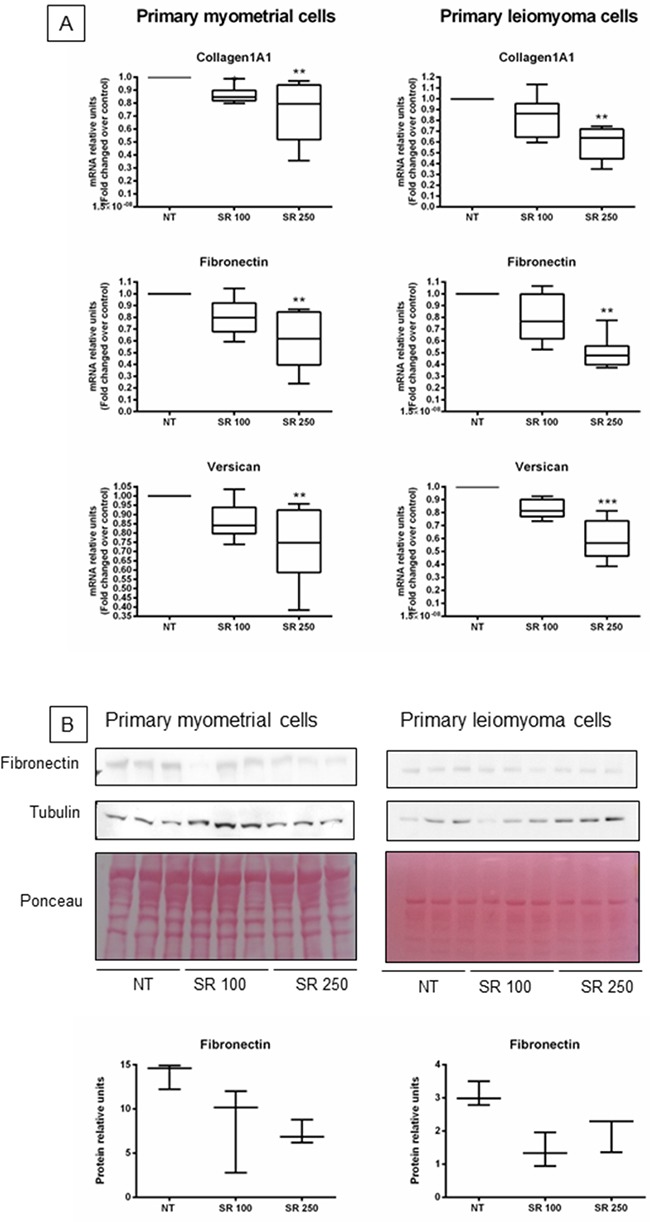
Effect of strawberry extract on extracellular matrix components expression in myometrial and leiomyoma cells **A**. mRNA expression. **B**. Protein expression. Data are expressed as mean ± SD (n=6 for RNA and n=3 for protein).NT, no treatment; SR 100, strawberry extract at 100 μg/ml; SR 250, strawberry extract at 250 μg/ml. *P<0.05; **P<0.01; ***P<0.001.

To determine the antifibrotic effect of strawberry extract, primary myometrial and leiomyoma cells were treated with strawberry extract (250 μg/ml) for 48 h, and measured mRNA and protein expression by real time PCR and western blot, respectively. We found that strawberry extract significantly reduced fibronectin, collagen 1A1, and versican mRNA expression in primary myometrial and leiomyoma cells compared with untreated cells (Figure 4A). We also found that protein expression of fibronectin was apparently reduced in strawberry treated myometrial and leiomyoma cells compared to untreated controls (Figure 4B).

### Effect of strawberry extract on activin A induced ECM expression in myometrial and leiomyoma cells

**Figure 5:**
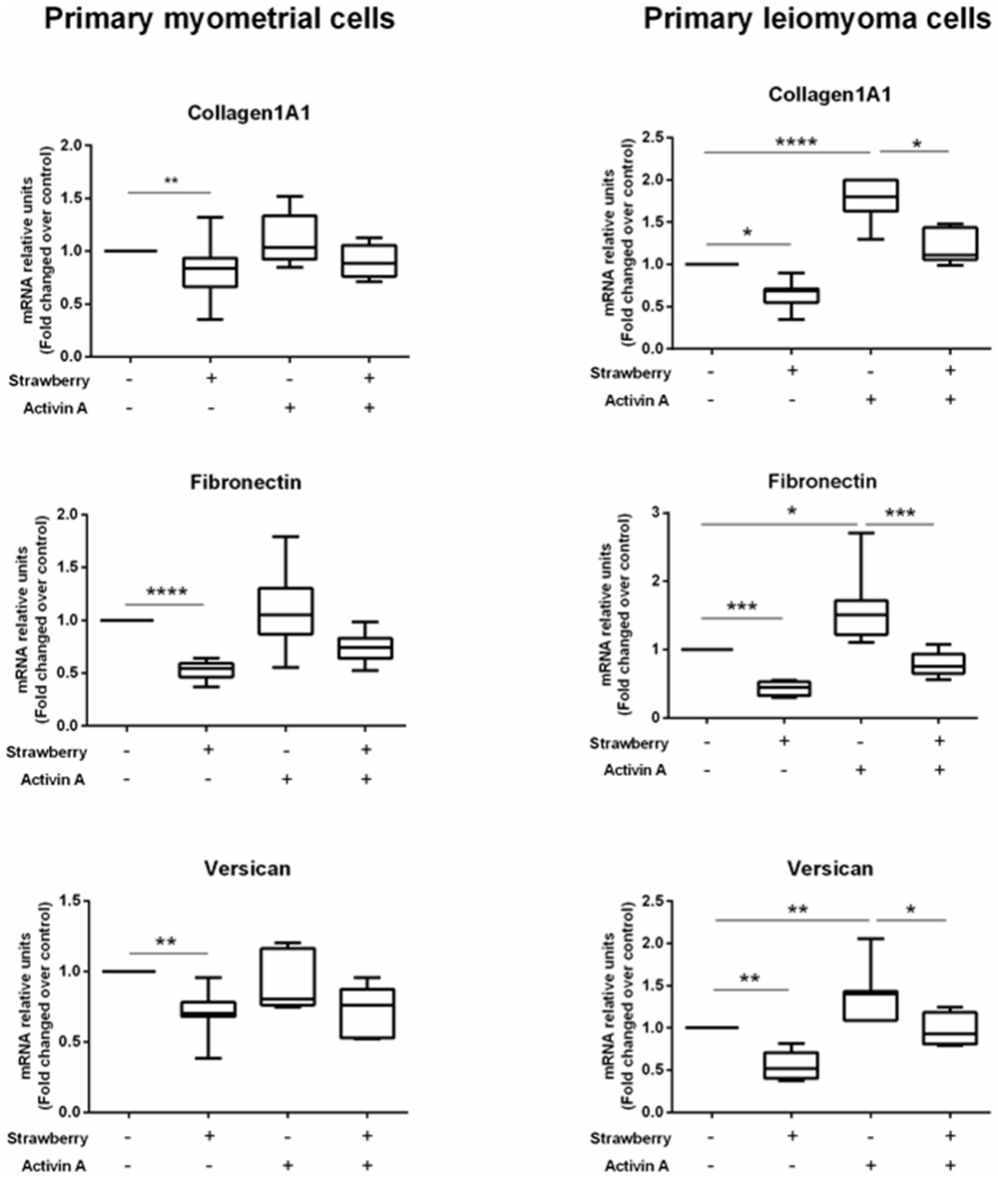
Effect of strawberry extract on activin A induced ECM components mRNA expression in myometrial and leiomyoma cells Data are expressed as mean ± SD (n=9). Strawberry extract at 250 μg/ml; activin A at 4 nM. *P<0.05; **P<0.01; ***P<0.001.

In a previous work, we demonstrated that activin A can increase collagen1A1, fibronectin and versican expression in primary leiomyoma cells [18]. Therefore, in this study, we investigated if strawberry extract can inhibit activin A induced ECM components expression in this cell type. We treated primary myometrial and leiomyoma cells with strawberry extract at 250 μg/ml for 48 h alone or activin A (4 nM) alone or combination of strawberry and activin A. We found that strawberry extract significantly reduced activin A induced fibronectin, collagen1A1, and versican mRNA expression in leiomyoma cells (Figure 5).

### Effect of strawberry extract on activin A and PAI-1 mRNA expression in myometrial and leiomyoma cells

**Figure 6:**
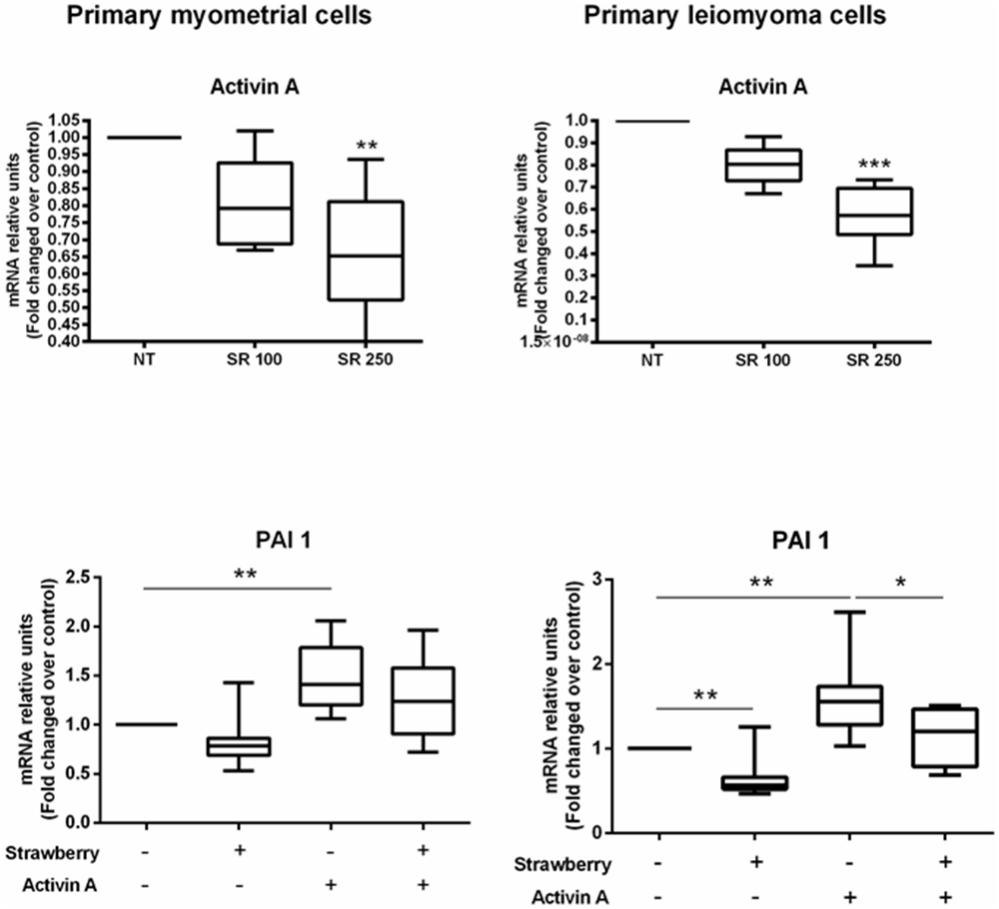
Effect of strawberry extract on activin A expression and activin A induced PAI-1 mRNA expression in myometrial and leiomyoma cells Data are expressed as mean ± SD (n=6 for activin A and n=9 for PAI-1). NT, no treatment; SR 100, strawberry extract at 100 μg/ml; SR 250, strawberry extract at 250 μg/ml; A, activin A at 4 nM. *P<0.05; **P<0.01; ***P<0.001.

After demonstrating that strawberry extract is able to directly inhibit ECM components as well as activin A induced ECM components expression in leiomyoma cells, therefore, we hypothesized that strawberry extract could inhibit ECM components by reducing activin A expression and/or by suppressing PAI-1 expression in leiomyoma cells. PAI-1 appears to play a significant role in the progression to fibrosis [19], and its expression is induced by activin A in mesangial cells [20]. We treated myometrial and leiomyoma cells with strawberry extract (250 μg/ml) for 48 h to measure activin A expression. To evaluate PAI-1 expression we treated myometrial and leiomyoma cells with activin A (4 nM) for 2 h in 48 h pre-treated with strawberry extract (250 μg/ml) or strawberry extract (250 μg/ml) alone for 48 h or activin A (4 nM) alone for 2 h. We found that strawberry extract significantly reduced activin A mRNA expression in both myometrial and leiomyoma cells (Figure 6). We also found that activin A was able to induce PAI-1 mRNA expression and that strawberry extract significantly inhibited activin A induced PAI-1 mRNA expression in leiomyoma cells (Figure 6).

## DISCUSSION

Although uterine leiomyoma is not a malignant disease, it is associated with several reproductive and gynecological problems, such as menorrhagia, dysmenorrhoea, chronic pelvic pain, infertility, recurrent miscarriage, preterm delivery and postpartum haemorrhage. In comparison with the burden of disease to women health, medical treatments are still limited and no preventative therapies have been developed. In this context, we introduce strawberry (a common fruit in human diet) for possible preventive and/or therapeutic option for uterine leiomyoma. The present study demonstrated the ability of strawberry to induce apoptosis and ROS production as well as decrease glycolysis and fibrosis in leiomyoma cells. These interesting results are highlighting the possible beneficial effect of strawberry for the management of uterine leiomyoma.

Apoptosis (also known as programmed cell death) is widely appreciated as a major mechanism of regulated cell death which plays a key role in embryogenesis, aging and tissue homeostasis. It is defined by distinct morphological and biochemical features, such as cell volume decrease, caspase activation, chromatin condensation, DNA fragmentation and finally, the breakdown of the cell into a series of smaller units (membrane-bound fragments). These are called apoptotic bodies and in most tissues are phagocytosed by adjacent cells [21]. The ability to modulate the life or death of a cell is recognized for its immense therapeutic potential. Consequently, many existing treatments (such as non-steroidal antiinflammatories and anticancer treatments) have been developed which may act through apoptosis [22]. Reactive oxygen species (ROS), such as superoxide anion, radicals, hydrogen and organic peroxides, are by-products of normal metabolism and xenobiotic exposure [23]. They can be beneficial or harmful to cells and tissues depending on their concentration. At physiological low levels, ROS can function as “redox messengers” in intracellular signaling and regulation. On the other hand, excess ROS can promote apoptosis and cell death [24]. In this study, we found that the ROS production and the percentage of apoptotic and dead cells were significantly higher in strawberry treated leiomyoma cells. This result suggests that the induction of cell death by strawberry may be due to overproduction of ROS. Additionally, ROS production was decreased in myometrial cells as well as no significant difference was observed in the percentage of apoptotic, dead and live cells in strawberry treated myometrial cells, suggesting the ability of strawberry extract to maintain homeostatic condition in normal cells.

Glycolysis is an anaerobic process that breaks down glucose and forms pyruvate with two ATPs in the cytoplasm. On the other hand, oxidative phosphorylation (OXPHOS) occurs in mitochondria and act as a major source of ATP in almost all aerobic organisms. Glycolysis and OXPHOS are tightly coupled to cooperate in maintaining the cellular energetic balance. In normal conditions, the cell metabolism consumes energy, of which 70% is supplied by OXPHOS. However, in hypoxia, a condition marked by an inadequate oxygen supply, glycolysis becomes enhanced to compensate for the weakened function of OXPHOS. Most cancer cells exhibit increased glycolysis and use this metabolic pathway to survive and growth [25]. Fibroid cells have been found to be severely hypoxic compared to the adjacent myometrium [26], suggesting an enhanced glycolytic activity in this cell type. The dependence of tumor cells on glycolytic pathway for ATP generation as a main source of their energy supply provides a biochemical basis for the design of therapeutic strategies to preferentially kill tumor cells by pharmacological inhibition of glycolysis. In the present study, we found that strawberry was able to decrease glycolytic rate in leiomyoma cells. In addition, both myometrial and leiomyoma cells showed no significant difference in OCR after strawberry treatment. This result suggests that strawberry may kill fibroid cells, at least in part, by inhibiting glycolysis.

One distinguishable character of uterine fibroids is the accumulation of excessive amount of extracellular matrix (ECM) components composed mainly of collagens, fibronectin, and versican [27–29]. Collagen is a central structural component of ECM known to maintain cellular morphology. It plays important roles in regulating proliferation, migration, differentiation, survival as well as wound healing and fibrotic process [30]. Leiomyoma cells demonstrated an overexpression of types I collagen mRNA in leiomyomas compared with the adjacent myometrium [27]. Fibronectin, a glycoprotein of the ECM, plays important roles in cell adhesion, migration, growth and differentiation as well as fibrosis [31]. Leiomyoma cells expressed elevated levels of fibronectin compared to myometrial cells [28]. Versican, a large chondroitin sulfate proteoglycan, plays an important role in cell migration, cell adhesion, cell proliferation, tissue stabilization, tissue homeostasis and inflammation [32, 33]. The up-regulation of versican expression was reported in leiomyoma cells compared to healthy counterparts [29]. The role of ECM proteins, in producing leiomyoma bulk structure and growth, provides an important way to control this tumor. Therefore, the development of antifibrotic agents could be a good solution for uterine fibroids. In the present study, we found that strawberry extract was able to inhibit ECM components, including collagen1A1, fibronectin, and versican in leiomyoma cells. This result suggests that strawberry could be used as a potential antifibrotic drug for treatment and/or prevention of uterine leiomyomas.

Activin A, a pleiotropic growth factor, belongs to the TGF-β superfamily, has been reported to increase ECM components, including collagen1A1, fibronectin, and versican mRNA expression in leiomyoma cells [18]. In addition to activin A, TGF-β3 was reported to increase collagen1A1, fibronectin 1 and versican expression in both myometrial and leiomyoma cells [28, 29, 34]. In the present study, we found that *Alba* cultivar of strawberry extract is able to inhibit activin A induced ECM collagen1A1, fibronectin, and versican mRNA expression in leiomyoma cells.

Finally, we investigated whether antifibrotic effect of strawberry extract is mediated by regulation of activin A expression or it can modulate PAI-1 expression, an important activin A-target. We found that strawberry extract significantly downregulates activin A mRNA expression in leiomyoma cells. In addition, activin A induces mRNA expression of PAI-1 in human uterine primary leiomyoma cells, which was inhibited by strawberry extract. PAI-1 is induced by activin A [20] and has been implicated in the pathology of fibrosis in different organs including, heart, lung, kidney, liver and skin [19]. The role of PAI-1 in leiomyoma fibrosis was demonstrated by the observation that TGF-β3 induced protein expression of PAI-1 in human uterine leiomyoma cells which was inhibited by antifibrotic compound, 1,25-Dihydroxyvitamin D3 [35]. The downregulation of activin A and PAI-1 expression by strawberry extract suggests that the antifibrotic effect of strawberry extract is mediated, at least in part, by regulation of activin A and PAI-1 expression.

Strawberry possesses a number of bioactive compounds such as, ellagic acid, quercetin, kaempferol, catechins, and anthocyanins [12] which are known to exert multiple therapeutic effects. The strawberry of cultivar Alba has been selected since it is a very interesting variety from the nutritional point of view, with a well-balanced micronutrient and phytochemical composition. In addition, in our previous studies, this cultivar showed a considerable health effect, thanks to its phenolic contents and its ability to maintain the cell membrane integrity, to reduce the free radical-dependent lipid peroxidation and to preserve and/or activate endogenous antioxidant enzymes [36, 37]. The ability of cultivar Alba strawberry extract to induce apoptosis and ROS, and decrease glycolysis and fibrosis in human uterine leiomyoma cells is may be due to the combination effect of phenolics and anthocyanins. Further studies need to be addressed to see the effect of single compounds in leiomyoma cells in comparison with strawberry extract.

In conclusion, the present study demonstrates that a phenolic and anthocyanin rich strawberry extract can induce apoptosis and ROS as well as suppress glycolysis and fibrosis in leiomyoma cells. These interesting results encourage further studies to highlight the bioactive compounds and the molecular mechanism involved, as well as to test the *in vivo* effects on uterine fibroids.

## MATERIALS AND METHODS

### Strawberry material

Ripe strawberry (*Fragaria × ananassa* Duch.) fruits from *Alba* cultivar were harvested from plants grown in an open experimental field of Azienda Agraria Didattico Sperimentale (Università Politecnica delle Marche, Ancona, Italy). Within 2 h after harvest, whole fruits were stored at -80°C for further analysis.

### Extract preparation

The hydroalcoholic extract was obtained from 10 g of fruits homogenized for 2 min in 100 ml (1:10 w/v) of extraction solution (80% methanol aqueous solution acidified with 0.1% formic acid) using an Ultra-turrax T25 homogenizer (Janke & Kunkel, IKA Labortechnik, Staufen, Germany). After homogenization the extraction was maximized by stirring the suspension for 2 h at 4°C in the dark. The mixture was then centrifuged at 1200 x g for 15 min (twice sequentially), to sediment solids, supernatants were filtered through a 0.45 μm Minisart filter (PBI International, Milan, Italy), transferred to 5 ml amber glass vials and stored at -80°C prior to analysis.

### Primary cell cultures

The study included samples of myometrial and leiomyoma tissue excised from women undergoing hysterectomy for fibroids. Considering the high variability that could occur with different age, race, hormonal milieu, tumor size, and location of tumors, we included in the study the most homogeneous sample possible. All patients were Caucasian (age range 41–49 yr) and in proliferative phase of the menstrual cycle. The location of the leiomyomas was intramural, and their size range was 7–10 cm in diameter. Patient's last menstrual period were used to determine the phase of the menstrual cycle. Tissue samples were only taken from women who had not received exogenous hormones for the previous 3 months. All patients gave their informed consent and the permission of the Human Investigation Committee was granted.

At the time of surgery, myometrial and leiomyoma samples were collected in Hanks’ Balanced Salt Solution (HBSS) (Euroclone, Milan, Italy), and immediately transported to the laboratory for necessary actions. Fibroid tissue was defined based on well-established histopathologic criteria. Samples were washed several times with Dulbecco's PBS (Invitrogen, Life Technologies, Carlsbad, CA, USA) to remove excess blood. After cutting tissue into small pieces, samples were mixed in 0.1% collagenase type 8 (Serva Electrophoresis GmbH, Heidelburg, Germany) in serum free Dulbecco Modified Eagle Medium (DMEM) (Lonza, Walkersville, MD, USA), and incubated at 37°C for 3-5 h in water bath with manual shaking. Digested cell suspensions were centrifuged at 1200 rpm for 10 min and then washed once with fetal bovine serum (FBS) (Gibco, Life Technologies). Finally, the cell pellet was dispersed in DMEM containing 10% FBS, 1% penicillin-streptomycin (EuroClone), 50 mg/L gentamicin (Lonza), and 1% Amphotericin B (Lonza), and plated in T25/T75 plastic dishes, and maintained using same media at 37°C in 95% air-5% CO2. The growth medium was changed after 48 h or 72 h to remove unattached cells and subsequently twice a week. The lower passage number (≤ P4) of cells was used for experiments to avoid changes in phenotype and gene expressions.

### Immunocytochemistry

The purity of cells was assessed by immunocytochemical staining with a specific smooth muscle cell marker, monoclonal mouse anti-α-smooth muscle actin (α-SMA) (Sigma-Aldrich, Milan, Italy). All cells were strongly positive for α-SMA (data not shown).

### Cell viability assay

Strawberry extract was concentrated under vacuum to eliminate total methanol and resuspended in fresh media to achieve different concentration (50, 100, 250, 500 μg/ml), as reported in our previous work [38]. To evaluate the potential cytotoxicity of the strawberry extract, cells were incubated with these different concentrations for 24h, 48h and 72h; control cells were incubated with only DMEM.

Cell viability was determined using the MTT assay, as previously described [39]. Results were expressed as a percentage of live cells compared to unexposed control. The data reported represent average values from at least three independent experiments.

### Image analysis assay for apoptosis detection

Apoptosis was measured in myometrial and leiomyoma cells using the Tali™ apoptosis assay kit and propidium iodide (Invitrogen™, Life Technologies) according to manufacturer's instructions. According to MTT results, cells were incubated with strawberry extract at 250 μg/ml for 48 h. At the end of incubation, cells were trypsinized, centrifuged (1500 rpm for 5 min), and resuspended in 100 μl of annexin binding buffer (ABB). After that, 5 μl of Annexin V Alexa Fluor® 488 was added, mixed well and the solution was incubated in dark at room temperature for 20 min. Then cells were centrifuged at 1500 rpm, resuspended in 100 μl of ABB and 1 μl of propidium iodide was added, mixed well and incubated in dark at room temperature for 5 min. Samples were analyzed using the Tali® Image-Based cytometer and the percentage of apoptotic nuclei, dead and live cells was determined on the basis of corresponding fluorescence histogram compared with untreated control. Apoptotic cells show green fluorescence, dead cells show red, and live cells show little or no fluorescence.

### Morphometric measurement of ROS levels

The determination of intracellular ROS levels was carried out using the probe CellROX® Orange reagent (Invitrogen, Life Technologies) according to manufacturer's instructions. After incubation of cells with strawberry extract (250 μg/ml) for 48 h, CellROX® Orange reagent (2 μl) was directly added to 1 ml of cells (1.5 × 105 cells/ml). Samples were incubated for 30 minutes at 37°C in water bath, centrifuged at 1500 rpm once to remove medium and excess dye and resuspended in HBSS. The image analysis of cells was performed with the Tali® Image-Based cytometer (Invitrogen, Life Technologies) collecting 20-fields per sample. Untreated cells were used to determine baseline levels of intracellular ROS and to set the fluorescence threshold for the Tali® instrument. The results were expressed as the percentage of cells with increased ROS levels compared with the control.

### Bioenergetic analysis

XF24 Extracellular Flux Analyzer from Seahorse Bioscience, Inc. (North Billerica, MA, USA) was utilized to detect oxygen consumption rate (OCR) and extracellular acidification rate (ECAR), representing oxidative phosphorylation (OXPHOS) and glycolysis, respectively, as previously described [40]. Briefly, leiomyoma and myometrial cells were seeded in 24-well XF cell culture microplates at 3.0×10^4^ cells/well for 16 hand then treated with strawberry extract (250 μg/ml) for 48 h. At the end of the treatment, the medium was replaced with 500 μl/well of XF-24 running media (supplemented with 25mM glucose, 2mM glutamine, 1mM sodium Pyruvate, without serum). The plates were pre-incubated at 37°C for 20 min in the XF Prep Station incubator (Seahorse Bioscience, Billerica MA, USA) in the absence of CO_2_ and then run on the XF24 analyzer to obtain OCR and ECAR. For each analysis, injections of different compounds, that modulate mitochondrial respiration or glycolysis, were injected sequentially, in each well: for OCR, oligomycin (5μg/ml), 2,4-DNP (150μM), rotenone (1μM), and antimycin A (1μM), while for ECAR rotenone (3 μM), glucose (10mM) and 2-DG (100mM). OCR and ECAR were recorded during specified programmed time periods (three readings each) as the average numbers between the injections of inhibitors mentioned above. The final data calculation was performed after the readings had been normalized with counting the cell number in each well. OCR and ECAR are expressed as pmol/min per 100000 cells and mpH unit change/min per 100000 cells, respectively.

### Real time PCR

Cells were lysed using TRIzol® reagent (Invitrogen, Life Technologies) and stored at -80°C. Total RNA (colorless upper aqueous phase) was isolated using chloroform according to the manufacturer's instructions. The ReliaPrep™ RNA Cell Miniprep System was used to purify and concentrate RNA (Promega Corp., Madison, WI, USA). We performed the reverse transcriptase (RT) using the high-capacity cDNA RT kit (Applied Biosystems, Life Technologies) with 1 μg RNA, and newly synthesized cDNA was used for real-time PCR. Real-time PCR was performed on StepOnePlus version 2.2.2 (Applied Biosystems, Life Technologies) in 96-well optical reaction plates with 50 ng cDNA in a final volume of 15 μl, containing 1X TaqMan® fast advanced master mix, with the following TaqMan® gene expression assays (Applied Biosystems, Life Technologies): collagen1A1 (Hs00164004_m1), fibronectin (Hs00365052_m1), versican (Hs00171642_m1), activin A (Hs00170103_m1), PAI 1 (Hs01126604_m1), HPRT (Hs99999909_m1) and ACTB (Hs99999903_m1). The blank for each reaction, consisting of amplifications performed in the absences of RT enzyme, was performed.

### Western blotting

At the end of treatment, cells were rinsed with PBS and solubilized using TRIzol® reagent (Invitrogen Life Technologies). Proteins were extracted following the manufacturer's instructions. Soluble protein was quantified using a Bradford protein assay (Bio-Rad, Milan, Italy), and equal amounts of proteins were loaded onto 4-12% NuPAGE gels (Life Technologies) and resolved by SDS-PAGE under reducing conditions. Proteins were transferred to 0.2-μm nitrocellulose membranes in an X-cell II apparatus (Invitrogen) according to the manufacturer's instructions. Ponceau S solution (Euroclone) was used for the detection of proteins on nitrocellulose membranes. After blocking the membrane with 5% (wt/vol) nonfat milk powder in Tris-buffered saline with Tween 20 (TBST) [50 mm Tris-HCl (pH 7.4), 150 mm NaCl, 0.05% Tween 20], membranes were incubated overnight with primary antibody as 1: 10000 dilutions for monoclonal mouse anti-human fibronectin (Sigma-Aldrich) or 1: 3000 dilutions for monoclonal mouse Tubulin (Sigma-Aldrich). Membranes were washed four times (10 min for each) in TBST and incubated with 1:1000 dilutions of horseradish peroxidase linked anti-mouse IgG (Amershan) against anti-human fibronectin and tubulin for 2 h. Membranes were washed four times (10 min for each) in TBST, and immunoreactive proteins were visualized using Super Signal West Pico chemiluminescent substrate (Pierce). The bands were quantified by scanning and analysis using Java-based image processing program, ImageJ 1.49n (National Institutes of Health, USA; http://imagej.nih.gov/ij).

### Data analysis

Statistical analyses were performed using GraphPad Prism version 6.01 for Windows (GraphPad, San Diego, CA). The data were analyzed using non-parametric ‘Kruskal-Wallis’ ANOVA, followed by post hoc ‘Dunn’ test for multiple comparisons. Results are expressed as mean±SD. Differences were considered significant when p < 0.05.

## References

[1] Islam MS, Protic O, Toti P, Giannubilo SR, Tranquilli AL, Petraglia F, Castellucci M, Ciarmela P (2013). Uterine leiomyoma: available medical treatments and new possible therapeutic options. J Clin Endocrinol Metab.

[2] Day Baird D, Dunson DB, Hill MC, Cousins D, Schectman JM (2003). High cumulative incidence of uterine leiomyoma in black and white women: ultrasound evidence. Am J Obstet Gynecol.

[3] Cramer SF, Patel A (1990). The frequency of uterine leiomyomas. Am J Clin Pathol.

[4] Cardozo ER, Clark AD, Banks NK, Henne MB, Stegmann BJ, Segars JH (2012). The estimated annual cost of uterine leiomyomata in the United States. Am J Obstet Gynecol.

[5] Stovall TG, Muneyyirci-Delale O, Summitt RL, Scialli AR (1995). GnRH agonist and iron versus placebo and iron in the anemic patient before surgery for leiomyomas: A randomized controlled trial. The Leuprolide Study Group. Obstet Gynecol.

[6] Stovall TG, Ling FW, Henry LC, Woodruff MR (1991). A randomized trial evaluating leuprolide acetate before hysterectomy as treatment for leiomyomas. Am J Obstet Gynecol.

[7] Islam MS, Akhtar MM, Ciavattini A, Giannubilo SR, Protic O, Janjusevic M, Procopio AD, Segars JH, Castellucci M, Ciarmela P (2014). Use of dietary phytochemicals to target inflammation, fibrosis, proliferation, and angiogenesis in uterine tissues: Promising options for prevention and treatment of uterine fibroids?. Mol Nutr Food Res.

[8] Zhang D, Al-Hendy M, Richard-Davis G, Montgomery-Rice V, Sharan C, Rajaratnam V, Khurana A, Al-Hendy A (2010). Green tea extract inhibits proliferation of uterine leiomyoma cells in vitro and in nude mice. Am J Obstet Gynecol.

[9] Ozercan IH, Sahin N, Akdemir F, Onderci M, Seren S, Sahin K, Kucuk O (2008). Chemoprevention of fibroid tumors by[−]-epigallocatechin-3-gallate in quail. Nutr Res.

[10] Roshdy E, Rajaratnam V, Maitra S, Sabry M, Allah ASA, Al-Hendy A (2013). Treatment of symptomatic uterine fibroids with green tea extract: a pilot randomized controlled clinical study. Int J Womens Health.

[11] Wise LA, Radin RG, Palmer JR, Kumanyika SK, Boggs DA, Rosenberg L (2011). Intake of fruit, vegetables, and carotenoids in relation to risk of uterine leiomyomata. Am J Clin Nutr.

[12] Giampieri F, Tulipani S, Alvarez-Suarez JM, Quiles JL, Mezzetti B, Battino M (2012). The strawberry: composition, nutritional quality, and impact on human health. Nutrition.

[13] Wang SY, Feng R, Lu Y, Bowman L, Ding M (2005). Inhibitory effect on activator protein-1, nuclear factor-kappaB, and cell transformation by extracts of strawberries (Fragaria× ananassa Duch.). J Agric Food Chem.

[14] Xue H, Aziz RM, Sun N, Cassady JM, Kamendulis LM, Xu Y, Stoner GD, Klaunig JE (2001). Inhibition of cellular transformation by berry extracts. Carcinogenesis.

[15] Seeram NP, Adams LS, Zhang Y, Lee R, Sand D, Scheuller HS, Heber D (2006). Blackberry, black raspberry, blueberry, cranberry, red raspberry, and strawberry extracts inhibit growth and stimulate apoptosis of human cancer cells in vitro. J Agric Food Chem.

[16] Zhang Y, Seeram NP, Lee R, Feng L, Heber D (2008). Isolation and identification of strawberry phenolics with antioxidant and human cancer cell antiproliferative properties. J Agric Food Chem.

[17] Diamanti J, Capocasa F, Balducci F, Battino M, Hancock J, Mezzetti B (2012). Increasing strawberry fruit sensorial and nutritional quality using wild and cultivated germplasm. PLoS One.

[18] Islam MS, Catherino WH, Protic O, Janjusevic M, Gray PC, Giannubilo SR, Ciavattini A, Lamanna P, Tranquilli AL, Petraglia F, Castellucci M, Ciarmela P (2014). Role of activin-A and myostatin and their signaling pathway in human myometrial and leiomyoma cell function. J Clin Endocrinol Metab.

[19] Ghosh AK, Vaughan DE (2012). PAI-1 in tissue fibrosis. J Cell Physiol.

[20] Gaedeke J, Boehler T, Budde K, Neumayer H-H, Peters H (2005). Glomerular activin A overexpression is linked to fibrosis in anti-Thy1 glomerulonephritis. Nephrol Dial Transplant.

[21] Fabisiak JP, Tyurina YY, Tyurin VA, Lazo JS, Kagan VE (1998). Random versus selective membrane phospholipid oxidation in apoptosis: role of phosphatidylserine. Biochemistry (Mosc).

[22] Nicholson DW (2000). From bench to clinic with apoptosis-based therapeutic agents. Nature.

[23] Fridovich I (1978). The biology of oxygen radicals. Science.

[24] Circu ML, Aw TY (2010). Reactive oxygen species, cellular redox systems, and apoptosis. Free Radic Biol Med.

[25] Pelicano H, Martin DS, RHa Xu, Huang P (2006). Glycolysis inhibition for anticancer treatment. Oncogene.

[26] Mayer A, Höckel M, Wree A, Leo C, Horn L-C, Vaupel P (2008). Lack of hypoxic response in uterine leiomyomas despite severe tissue hypoxia. Cancer Res.

[27] Stewart EA, Friedman AJ, Peck K, Nowak RA (1994). Relative overexpression of collagen type I, collagen type III messenger ribonucleic acids by uterine leiomyomas during the proliferative phase of the menstrual cycle. J Clin Endocrinol Metab.

[28] Arici A, Sozen I (2000). Transforming growth factor-beta3 is expressed at high levels in leiomyoma where it stimulates fibronectin expression, cell proliferation. Fertil Steril.

[29] Norian JM, Malik M, Parker CY, Joseph D, Leppert PC, Segars JH, Catherino WH (2009). Transforming growth factor beta3 regulates the versican variants in the extracellular matrix-rich uterine leiomyomas. Reprod Sci.

[30] Pickering JG (2001). Regulation of vascular cell Behavior by collagen form is function. Circ Res.

[31] Pankov R, Yamada KM (2002). Fibronectin at a glance. J Cell Sci.

[32] Wight TN (2002). Versican: a versatile extracellular matrix proteoglycan in cell biology. Curr Opin Cell Biol.

[33] Andersson-Sjöland A, Hallgren O, Rolandsson S, Weitoft M, Tykesson E, Larsson-Callerfelt A-K, Rydell-Törmänen K, Bjermer L, Malmström A, Karlsson JC (2014). Versican in inflammation and tissue remodelling: the impact on lung disorders. Glycobiology.

[34] Joseph DS, Malik M, Nurudeen S, Catherino WH (2010). Myometrial cells undergo fibrotic transformation under the influence of transforming growth factor beta-3. Fertil Steril.

[35] Halder SK, Goodwin JS, Al-Hendy A (2011). 1,25-Dihydroxyvitamin D reduces TGF-β3-induced fibrosis-related gene expression in human uterine leiomyoma cells. J Clin Endocrinol Metab.

[36] Alvarez-Suarez J, Dekanski D, Ristić S, Radonjić NV, Petronijević NaD, Giampieri F, Astolfi P, González-Paramás AM, Santos-Buelga C, Tulipani S, Quiles JL, Mezzetti B, Battino M (2011). Strawberry polyphenols attenuate ethanol-induced gastric lesions in rats by activation of antioxidant enzymes and attenuation of MDA increase. PLoS One.

[37] Alvarez-Suarez JM, Giampieri F, Tulipani S, Casoli T, Di Stefano G, González-Paramás AM, Santos-Buelga C, Busco F, Quiles JL, Cordero MD, Bompadre S, Mezzetti B, Battino M (2014). One-month strawberry-rich anthocyanin supplementation ameliorates cardiovascular risk, oxidative stress markers and platelet activation in humans. J Nutr Biochem.

[38] Giampieri F, Alvarez-Suarez JM, Mazzoni L, Forbes-Hernandez TY, Gasparrini M, Gonzalez-Paramas AM, Santos-Buelga C, Quiles JL, Bompadre S, Mezzetti B (2014). An anthocyanin-rich strawberry extract protects against oxidative stress damage and improves mitochondrial functionality in human dermal fibroblasts exposed to an oxidizing agent. Food & function.

[39] Maines MD, Costa LG, Reed DJ, Sassa S, Sipes IG (1998). Current protocols in toxicology.

[40] Somasagara RR, Deep G, Shrotriya S, Patel M, Agarwal C, Agarwal R (2015). Bitter melon juice targets molecular mechanisms underlying gemcitabine resistance in pancreatic cancer cells. Int J Oncol.

